# Changes in Pancreatic Cancer Management and Surgical Treatment During the COVID-19 Pandemic

**DOI:** 10.3390/medicina60121924

**Published:** 2024-11-22

**Authors:** Aida Puia, Catalin Vladut Ionut Feier, Vasile Gaborean, Raluca Bodea, Florin Graur, Florin Zaharie, Nadim Al-Hajjar, Ion Cosmin Puia

**Affiliations:** 1 Department of Community Medicine, “Iuliu Hațieganu” University of Medicine and Pharmacy, 400347 Cluj-Napoca, Romania; draidapuia@gmail.com; 2 First Discipline of Surgery, Department X-Surgery, “Victor Babes” University of Medicine and Pharmacy, Eftimie Murgu Square No. 2., 300041 Timisoara, Romania; 3 First Surgery Clinic, “Pius Brinzeu” Clinical Emergency Hospital, 300723 Timisoara, Romania; 4 Department of Surgical Semiology, Faculty of Medicine, “Victor Babeş” University of Medicine and Pharmacy Timisoara, Eftimie Murgu Square No. 2, 300041 Timişoara, Romania; vasile.gaborean@umft.ro; 5 Regional Institute of Gastroenterology and Hepatology “Octavian Fodor”, 400394 Cluj-Napoca, Romania; ralucabodea@yahoo.com (R.B.); florin.graur@umfcluj.ro (F.G.); zaharie.vasile@umfcluj.ro (F.Z.); na_hajjar@yahoo.com (N.A.-H.); drpuia@yahoo.fr (I.C.P.); 6 Department of Surgery, “Iuliu Hatieganu” University of Medicine and Pharmacy, 400012 Cluj-Napoca, Romania

**Keywords:** pancreatic ductal adenocarcinoma, COVID-19, surgery, postoperative outcomes, hospitalization, postoperative complications, neoadjuvant therapy

## Abstract

*Background and Objectives*: This study evaluated the impact of the COVID-19 pandemic on the surgical management of pancreatic ductal adenocarcinoma (PDAC) at a tertiary care hospital in Romania. The objective was to compare surgical volumes, tumor characteristics, and patient outcomes across three periods: pre-COVID, pandemic, and post-COVID. *Materials and Methods*: A retrospective analysis of 622 PDAC patients who underwent surgery between February 2018 and February 2024 was conducted. The key variables analyzed included tumor size, type of surgery (curative vs. palliative), use of neoadjuvant therapy, postoperative complications, and ICU monitoring, among others. *Results*: During the pandemic, there was a 25% decrease in surgical interventions compared the number performed during the pre-pandemic period, with a significant increase in the number of patients undergoing surgical intervention following neoadjuvant treatment (*p* = 0.009) in the post-pandemic period. Post-pandemic, surgical volumes increased by 10%, and tumor sizes were smaller (*p* = 0.029). Postoperative outcomes, such as complications, remained stable across the periods, but intensive care unit monitoring increased significantly during the pandemic and post-pandemic periods. Hospital stay durations were significantly shorter during and after the pandemic (*p* < 0.05). *Conclusions*: The COVID-19 pandemic led to delays in PDAC surgeries, but post-pandemic improvements in surgical volumes and early diagnosis are evident; however, further optimization of screening and treatment protocols is essential for improving patient outcomes.

## 1. Introduction

Pancreatic ductal adenocarcinoma (PDAC) is the most common type of pancreatic cancer, accounting for over 90% of cases [1]. It is a particularly aggressive malignancy with a poor prognosis, often diagnosed at an advanced stage due to its asymptomatic nature in early development. The five-year survival rate remains below 10% globally, making it one of the most lethal cancer types [2].

The incidence of PDAC has been on the rise, and it currently ranks as the 12th most common cancer worldwide, with an estimated 458,918 new cases diagnosed in 2020 [3]. Risk factors include smoking, chronic pancreatitis, diabetes, obesity, and genetic predispositions [4]. Early diagnosis and prompt treatment are critical for improving outcomes, but these have been significantly challenged by the COVID-19 pandemic, which disrupted regular screening and healthcare services.

The COVID-19 pandemic has introduced significant challenges into the management of pancreatic ductal adenocarcinoma. Lockdowns and healthcare system constraints led to delays in diagnosis, postponement of surgeries, and interruptions in chemotherapy regimens [5]. Studies have reported a shift toward neoadjuvant chemotherapy to delay surgeries and reduce hospital visits, potentially affecting long-term survival outcomes in oncological patients [6,7]. Although neoadjuvant chemotherapy is an effective interim solution for PDCA [8], changes in treatment protocol have not yet proven to be equally effective [9]. 

This retrospective study assesses the impact of the COVID-19 pandemic on the clinical presentation and surgical management of PDAC patients at the Prof. Dr. Octavian Fodor Regional Institute of Gastroenterology and Hepatology in Cluj-Napoca, Romania, by comparing patient data from the pre-pandemic, pandemic, and post-pandemic periods. It also examines post-pandemic changes in healthcare services, highlighting the ongoing effects of the global crisis on the return to standard practices.

## 2. Materials and Methods

### 2.1. Study Type, Timeline, and Site

This study gathers data from 622 patients undergoing surgical intervention for PDAC between 26 February 2018 and 25 February 2024 at the Prof. Dr. Octavian Fodor Regional Institute of Gastroenterology and Hepatology in Cluj-Napoca, Romania.

Before undergoing surgical intervention for the treatment of pancreatic cancer, each case was discussed in a multidisciplinary committee to determine the appropriate therapeutic approach. Patients with resectable tumors were directed towards surgical treatment, while those with borderline resectable tumors were guided towards neoadjuvant therapy, with the intention of returning for surgical intervention afterward.

To elucidate the influence of the COVID-19 pandemic on the treatment and management of these patients, the study period was stratified into three distinct phases:

- 26 February 2018 to 25 February 2020, representing the pre-COVID group.
- 26 February 2020 to 25 February 2022, representing the pandemic group.
- 26 February 2022 to 25 February 2024, representing the post-COVID group.

On 26 February 2020, Romania reported its first confirmed case of COVID-19, marking the beginning of the pandemic in the country. Subsequently, on 8 March 2022, all government-imposed restrictions were lifted. 

### 2.2. Inclusion Criteria

Following the determination of the study period, the inclusion criteria were carefully designed to ensure the selection of an appropriate and representative study population. The inclusion criteria are as follows:

- Undergoing either palliative or curative surgical intervention for the treatment of PDAC.
- Surgical treatment performed within the specified time period.
- The presence of a postoperative histopathological diagnosis of PDAC.

Considering the aim of this study, additional inclusion criteria were implemented to ensure a thorough assessment. It is well established that an active SARS-CoV-2 infection can significantly affect the prognosis and postoperative outcomes in patients with PDAC [10,11]. One of the primary objectives of this study was to illustrate how the surgical treatment of this pathology was impacted during the pandemic, rather than focusing on the infection itself or its direct consequences regarding PDAC patients. 

The following inclusion criteria were added:

- Absence of SARS-CoV-2 infection either before surgery or during hospitalization.
- Absence of any typical COVID-19 symptoms upon admission or within the seven days preceding admission.
- Obtaining a negative reverse transcription polymerase chain reaction (RT-PCR) test result for SARS-CoV-2 at the time of admission or within 24 h prior to admission.

### 2.3. Analysis of Clinical Variables and Surgical Data

Once the inclusion criteria were met, multiple parameters for these patients were investigated, including data such as gender, age, and environment of origin. The Results section outlines the tumor locations in different segments of the pancreas, including the head, body, tail, and corporeo-caudal regions. Additionally, the types of interventions performed during the three study periods were analyzed and categorized into two groups: palliative and curative.

The curative interventions performed were as follows:

- Pancreaticoduodenectomy (Whipple procedure);
- Distal pancreatectomy with splenectomy;
- Central pancreatectomy;
- Total pancreatectomy with duodenectomy;
- Other surgical procedures (curative interventions performed involved high complexity with multiple organ resections).

The palliative interventions performed were as follows:

- Choledochoduodenostomy;
- Choledochojejunostomy;
- Hepaticojejunostomy;
- Gastrojejunostomy;
- Other surgical procedures (palliative interventions involving more than one palliative procedure).

Postoperative complications, such as fistula or hemorrhage, were analyzed using the Clavien–Dindo classification. Additionally, the proportion of patients who underwent surgical treatment after neoadjuvant chemotherapy during the three study periods was also analyzed. 

The necessity of postoperative hospitalization in the ICU (intensive care unit), as well as the duration of time patients spent in the ICU, were also analyzed. The patients’ comorbidities were assessed using the Charlson Comorbidity Index. Tumor size (longest tumor axis dimension following histopathological examination), along with the stage of tumor invasion (T), lymph node involvement (N), the presence or absence of metastasis (M), and consequently, the overall stage of the disease, were also taken into consideration.

In addition, due to the unique circumstances generated by this pandemic and the risk of SARS-CoV-2 infection associated with prolonged exposure to the medical environment, we also analyzed the total duration of hospitalization, along with preoperative and postoperative hospital stay durations and the duration of surgery. Lastly, postoperative mortality was also analyzed.

### 2.4. Statistical Analysis

For statistical analysis, we used IBM SPSS Statistics Version 25 software for Windows (IBM Corp., Armonk, NY, USA). Descriptive statistics were applied to numerical variables, including the computation of central tendency measures and dispersion indices. For categorical variables, frequency distributions and percentage values were used to depict variations across the study periods.

To assess whether the numerical data followed a normal distribution, the Shapiro–Wilk test was employed, with a *p*-value of less than 0.05 indicating a deviation from normality. For comparing two independent groups, the Mann–Whitney U test was used, or the Student’s t-test was applied when the variables were normally distributed. The Kruskal–Wallis H test was employed for comparisons involving more than two groups when the variables did not follow a normal distribution. For data that met the assumption of normal distribution, the ANOVA test was utilized to assess variance across multiple categories, enabling a more robust comparison of group means.. The Chi-square test was employed to examine associations between categorical variables and to identify significant differences in proportions.

Statistical significance was set at *p* < 0.05 for all tests, indicating that the observed results are unlikely to be due to random variation.

## 3. Results

### 3.1. Patient Demographics

Once the inclusion criteria were met, data from 622 patients with PDAC who underwent surgical intervention at the institute were analyzed. Out of these, 181 patients (29.1%) were treated during the pandemic, 242 patients (38.9%) underwent surgical treatment during the pre-COVID period, and 199 patients (32%) underwent surgery in the post-COVID period. Demographic data, along with variations in the Charlson Comorbidity Index (>3), did not show statistically significant variation across the three periods. The results are presented in Table 1.

The mean variation of the Charlson Comorbidity Index between the pandemic period and the first study period was analyzed. A variation in the mean value was observed, from 5.19 ± 2.18 in the first period to 4.70 ± 1.9 during the pandemic period (*p* = 0.049).

### 3.2. Tumor Characteristics

Variations of tumor location and the average dimension are presented in Table 2.

Analyzing the post-COVID period individually compared to the pre-COVID period, in terms of the mean tumor size, the results were 3.3 ± 1.32 vs. 2.98 ± 1.08, showing a *p*-value = 0.029,.

The variation in T-stage, along with the stage of pancreatic cancer, are presented in Table 3.

Table 4 illustrates the variation in lymph node involvement among patients who underwent curative surgery across the three periods.

Regarding the variation in the N stage between the pandemic and post-pandemic periods, a *p*-value of 0.015 was obtained. 

The proportion of curative and palliative surgical interventions was also evaluated. In the first study period, 126 curative interventions (52.1%) were performed, during the pandemic 117 patients (64.6%) underwent curative treatment, and in the final period, 138 patients (69.3%) were treated surgically (*p* = 0.001). 

The variation in curative surgical techniques applied over the three periods is presented in Table 5.

The variation in palliative surgical techniques applied over the three periods is presented in Table 6.

The proportion of patients who received preoperative neoadjuvant chemotherapy was analyzed. Over the three periods, a total of 34 patients received this treatment before surgery: 8 in the first period, 7 in the second, and 19 in the final period (*p* = 0.009). 

### 3.3. Postoperative Outcomes and Complications

A total of 92 patients experienced postoperative complications. Seven patients presented with both fistula and hemorrhage. During the pre-COVID period, 34 patients (36.69%) experienced complications, 27 patients (29.03%) suffered complications during the pandemic, and 31 patients (33.69%) in the final study period. The incidence of postoperative complications was notably higher among patients who underwent extensive curative interventions. Of the 92 total cases of complications, 85 were observed in the cohort of patients receiving curative treatments (*p* < 0.001). The distribution of postoperative complications in these patients, as categorized by the Clavien–Dindo classification, is detailed in Table 7.

Regarding postoperative ICU hospitalization, it was necessary in 131 cases (54.1%) during the pre-pandemic period, while 124 patients (68.5%) required at least one day of ICU monitoring during the pandemic. In the final period, this number increased to 159 patients (79.9%). After identifying differences in proportions across the three periods concerning the need for postoperative ICU monitoring, a *p*-value of <0.001 was obtained.

Analysis of the postoperative ICU stay duration revealed a mean of 5.92 ± 10.87 days in the pre-COVID period, 5.62 ± 7.88 days during the pandemic, and 6.03 ± 6.55 days in the post-COVID period (*p* = 0.922). 

Within the study, 36 patients died postoperatively. In the first period, 11 patients (4.5%) died, 11 patients (6.1%) died during the pandemic period, and 14 patients (7%) died in the post-COVID period (*p* = 0.527). 

Regarding the mean variations in the duration of the surgical intervention, preoperative hospital stay, and postoperative hospital stay, the tests applied generated *p*-values of <0.05, highlighting statistically significant differences between the three periods. These results are presented in Table 8.

## 4. Discussion

This study revealed several important findings regarding the impact of the COVID-19 pandemic on the surgical treatment of PDAC. Firstly, there was a notable 25% decrease in surgical interventions during the pandemic, reflecting the global trend of decreased elective surgeries in oncological care. However, post-pandemic data showed a 10% increase in surgical volume, indicating a gradual recovery of healthcare services and a return to pre-pandemic activity levels. Furthermore, tumor sizes were notably smaller in the post-pandemic period, indicating potential advancements in early diagnosis, likely attributed to improved screening protocols and heightened patient awareness in the aftermath of the pandemic. Moreover, hospital stay durations, both preoperative and postoperative, were significantly reduced during and after the pandemic, an outcome likely driven by the need to minimize patient exposure to COVID-19 and streamline hospital workflows for efficiency and safety.

The COVID-19 pandemic represented a global health crisis that severely affected healthcare systems worldwide [7,12]. The management of surgical pathologies was profoundly impacted, with a significant reduction in the number of procedures, particularly during the initial phase of the pandemic. For oncological conditions, which are already associated with a challenging prognosis, delays in surgical intervention can have substantial adverse effects on both medium- and long-term patient outcomes. According to a study by Bennet S. et al., for every month of delay in surgical treatment, the risk of death increases by approximately 6–13%, depending on the type of cancer, including pancreatic cancer [13]. Romania was no exception to this scenario, and this study aims to present how the pandemic period affected the management and surgical treatment of patients with PDAC at the Prof. Dr. Octavian Fodor Regional Institute of Gastroenterology and Hepatology, a tertiary hospital in Cluj-Napoca, Romania.

Various studies have highlighted the impact of the pandemic on surgical treatment for oncological pathologies in Romania, with results similar to those observed worldwide [14,15,16]. This was primarily due to the postponement of surgical interventions, as initially recommended by various surgical societies, including the American College of Surgeons (ACS) [17], the Society of American Gastrointestinal and Endoscopic Surgeons (SAGES) [18], and national health authorities. Digestive oncological pathologies were no exception, with significant changes reported in the number of surgeries performed, patients’ postoperative outcomes, and the manner of their presentation [19,20,21,22].

In addition to the restrictions imposed by authorities, advising patients to seek hospital care only for emergencies, the fear of contracting the novel coronavirus, coupled with psycho-emotional stressors, led to a significant decline in hospital visits. Consequently, patients frequently presented to healthcare facilities only when their symptoms had progressed to conditions that were extremely difficult to tolerate and manage. Byrnes et al. [23] describe how fear of COVID-19 delayed necessary surgical interventions. Meanwhile Lazzerini et al. report that fear and anxiety surrounding the virus resulted in delayed care and reduced hospital visits, even in cases of severe symptoms [24].

In patients with PDAC, this study observed a 25% decrease in surgical interventions during the two years of the pandemic compared to during the pre-COVID period, followed by a 10% increase in the post-COVID period. This significant reduction in surgical procedures aligns with findings from a study conducted in the United Kingdom, which reported a 36% decrease in elective surgeries for pancreatic cancer during the early stages of the pandemic [25]. A global analysis estimated that approximately 28 million elective surgeries were postponed in 2020, including essential oncological procedures [26].

One of the significant challenges of PDAC is its late diagnosis, where screening plays a crucial role. Globally, pancreatic cancer screening is not widely implemented; however, several studies conducted during the pandemic reported a substantial decline in screening rates across European countries, including Italy [27], Germany, and the UK [28], with reductions reaching up to 60%. The United States experienced a 50% decrease in screening rates, while Canada observed a 55% reduction [29]. Similarly, in Asia, the pandemic had a significant impact, with Singapore—a leader in advanced technology and healthcare—reporting a 40% decline [30]. Following the end of restrictions and the reopening of healthcare services, screening rates have shown signs of recovery, and emotional factors have contributed to a greater awareness of the aggressive nature of oncological diseases, encouraging patients to seek medical care more proactively [31,32].

In our study, the demographic data did not differ significantly across the three study periods. The Charlson Comorbidity Index showed no significant variations between the periods; however, a more detailed analysis of the mean variation during the pandemic period revealed a significant increase. It is clear that during the pandemic, patients presenting with milder symptoms and generally better overall health were more likely to avoid hospital visits. Consequently, those who did seek medical care presented with more severe symptoms and higher comorbidity levels, often waiting until their condition became critical. A similar situation was reported in a study from the United Kingdom, where oncological patients diagnosed during the pandemic exhibited, on average, larger tumors and a higher number of comorbidities compared to those diagnosed pre-pandemic. The Charlson Comorbidity Index increased by 18% during the pandemic, indicating later presentations and a generally poorer health status at the time of diagnosis [21].

Although there was no significant variation in the primary tumor location across the three study periods, a statistically significant decrease in tumor size was observed, particularly between the pre-COVID and post-COVID groups (*p* = 0.029). This reflects that the pandemic led to an increased awareness among patients and improvements in the healthcare system, resulting in higher screening rates and earlier detection post-pandemic [31,32].

During the pandemic, there were widespread discussions and recommendations to delay surgical interventions and to direct patients toward neoadjuvant therapy, mainly due to the additional risks posed by postoperative COVID-19 infection, which had known devastating effects [10,11]. It is well established that neoadjuvant treatment is essential for patients with borderline resectable tumors in PDAC [8]. The increase in the number of such cases indicates that delays in seeking medical care have led to a higher incidence of patients presenting with borderline resectable tumors. In our study, there was a statistically significant increase in the number of patients receiving neoadjuvant therapy (*p* = 0.009). In the post-COVID period, 19 patients underwent surgery following neoadjuvant treatment, compared to 8 and 7 in the other two periods. Although neoadjuvant treatment is typically the initial step in managing borderline resectable pancreatic cancer, during the pandemic, many medical centers worldwide adopted neoadjuvant therapies as a strategy for resecable tumors as well in order to postpone surgeries until the epidemiological situation stabilized, as confirmed by the literature [33,34,35,36].

When analyzing the types of surgical interventions performed (radical/palliative), a statistically significant difference was observed across the three periods (*p* = 0.001). Specifically, the number and proportion of curative surgeries increased over time. This shift can be attributed to the prioritization of oncological care during the pandemic, with patients presenting higher chances of curative treatment being prioritized, while those with advanced disease were directed toward palliative care or neoadjuvant treatment. Examining the variation in techniques used for the two types of interventions, a significant shift was noted in palliative techniques, with a decrease in coledocoduodenostomies and an increase in gastrojejunostomies, particularly in the post-pandemic period (*p* < 0.001). This approach is supported by Bennett et al., who emphasized that surgical interventions were prioritized for oncological patients with better survival prospects, while those with more advanced disease were directed toward palliative treatments or close monitoring [13]. Conversely, Glehen et al. highlighted a significant increase in the use of palliative techniques in the post-pandemic period, largely due to patients presenting with more advanced pathology as a result of delayed hospital visits and associated psycho-emotional factors [37].

Surgical interventions for PDAC are not immune to postoperative complications, with anastomotic fistulas and postoperative hemorrhages being the most notable. The occurrence of these complications is influenced by various factors, including patient status; associated comorbidities; and metabolic, hematological, hydroelectrolytic, and protein imbalances. This study presents a variation in postoperative complications comparable to those noted in the literature, and the proportion of patients experiencing complications did not significantly change across the three periods. Thus, we can hypothesize that the COVID-19 pandemic did not present an additional risk in this regard. According to recent studies, the proportion of complications did not vary significantly over the pandemic [12,38]. For example, the studies by Chon et al. [39] and Madge et al. [40] demonstrated that patients with PDAC treated during the pandemic showed complication rates comparable to those for the pre-pandemic periods.

However, the proportion of patients requiring postoperative ICU monitoring increased significantly over the three periods. Since the onset of the pandemic, postoperative monitoring has become more meticulous, with a focus on quicker patient rebalancing and the early identification of adverse outcomes to enable prompt intervention and stabilization. This approach aimed to reduce both hospital stay duration and the risk of COVID-19 exposure. Bennett et al. reported that this type of intensive monitoring allowed hospitals to shorten hospitalization time without compromising patient safety [13].

As previously anticipated, one of the major concerns during the pandemic was the increased risk of contracting COVID-19 within the hospital environment, a high-risk setting. As mentioned in the Materials and Methods section, all patients admitted to the institute were tested for COVID-19 upon admission and isolated until receiving the RT-PCR results. In the case of a positive result, patients were directed to a specialized ward, or if they were asymptomatic, they were isolated at home. This study showed a significant reduction in total hospital stay for both the preoperative and postoperative periods, as the clinic strictly avoided unnecessary hospitalizations. This reduced hospital stay was, of course, contingent upon a favorable postoperative evolution—once patients were hemodynamically stable, afebrile, had regained appetite, and bowel function had resumed, they were discharged. The shorter postoperative recovery was supported by intensive ICU monitoring, which allowed for early rebalancing and rapid intervention. Gonzalez-Montero et al. [41] noted that reducing hospital stays was a preventive measure against SARS-CoV-2 infection, while ensuring safe and rapid patient discharge after hemodynamic stabilization. Similarly, Glehen et al. reported that shorter hospital stays were associated with careful ICU monitoring and early rebalancing of patients, reducing their exposure to infection risks [37].

After this pandemic, oncological management will need to adapt, not only by focusing on recovering the volume of surgical interventions but also by developing more effective and accessible screening strategies. These changes highlight the need for the post-pandemic optimization of medical services to address the gaps in diagnosis and treatment and to mitigate long-term negative effects.

### Study Limitations

Our study has several significant limitations. First, as it was conducted at a single tertiary center, the results cannot be generalized to the national or international level. A larger study incorporating data from multiple centers would provide a clearer perspective of the impact of the pandemic on patients with PDAC. Additionally, we lack information on patients who may have died from COVID-19 or other causes, but who had undiagnosed PDAC, suggesting the possibility of underdiagnosis. It is important to recognize the potential for outcome misclassification, particularly regarding tumor staging (T-stage, N-stage and M-stage). Differences in interpretations by various healthcare professionals may have contributed to misclassification, potentially affecting the perceived clinical relevance of the analyzed ratios. Additionally, these patients were operated on by multiple surgeons, which could lead to variability in postoperative outcomes. This variability may be primarily attributed to differences in each surgeon’s level of experience in treating this pathology, as well as to their individual surgical techniques, approaches, and preferences in specific steps of the procedure.

Moreover, some patients did not seek hospital care for various reasons, but they may have had PDAC, which distorts the data regarding the true incidence of the disease. Although the study outlines a return to normalcy in certain parameters during the post-pandemic period, the long-term impact of delayed diagnosis and treatment on survival remains uncertain. However, a strength of this study is that it provides a detailed surgical evolution recovery in the post-pandemic era, indicating a positive adaptation in patient management.

## 5. Conclusions

This study evaluated the impact of the COVID-19 pandemic on the surgical treatment of PDAC across three distinct periods. Post-pandemic, surgical volumes partially recovered, and tumor sizes were smaller, indicating improvements in early diagnosis. Postoperative outcomes, including complications, remained stable, although more patients required intensive care during the pandemic. Hospital stays were shortened during and after the pandemic, likely due to efforts to reduce COVID-19 exposure.

The pandemic significantly disrupted PDAC care, but healthcare systems adapted by optimizing surgical schedules, enhancing patient triage processes, and improving postoperative care to mitigate the impact of delayed treatments. Moving forward, refining screening and treatment strategies will be crucial for improving patient outcomes and addressing pandemic-related delays.

## Figures and Tables

**Table 1:** Demographic data.

Variables	Pre-Pandemic n = 242	Pandemic n = 181	Post-Pandemic n = 199	*p*
Age (years, M ± SD)	65.82 ± 8.78	65.27 ± 9.22	66.78 ± 8.64	0.242 ^a^
Gender				
Male	128 (52.9%)	85 (47%)	99 (49.7%)	0.478 ^b^
Female	114 (47.1%)	96 (53%)	100 (50.3%)	
Environment				
Urban	156 (64.5%)	104 (57.5%)	122 (61.3%)	0.342 ^b^
Rural	86 (35.5%)	77 (42.5%)	77 (38.7%)	
Charlson > 3	174 (71.9%)	127 (70.2%)	133 (66.8%)	0.510 ^b^

M = mean; SD = standard deviation. ^a^ ANOVA test; ^b^ Chi-square test.

**Table 2:** Tumor characteristics.

Variables	Pre-Pandemic	Pandemic	Post-Pandemic	*p*
Tumor location				
Pancreatic head	219 (90.5%)	158 (87.3%)	177 (89%)	0.388 ^b^
Pancreatic body	11 (4.5%)	14 (7.7%)	9 (4.5%)	
Pancreatic tail	2 (0.8%)	5 (2.8%)	5 (2.5%)	
Corporeo-caudal	10 (4.1%)	4 (2.2%)	8 (4%)	
Tumor size (cm, M ± SD)	3.3 ± 1.32	3.08 ± 1.25	2.98 ± 1.08	0.087 ^a^

M = mean; SD = standard deviation; cm = centimeters; ^a^ ANOVA test; ^b^ Chi-square test.

**Table 3:** T-stage and cancer stage variation.

Variables	Pre-Pandemic	Pandemic	Post-Pandemic	*p*
T1a	0	1 (0.6%)	1 (0.5%)	0.033 ^b^
T1b	0	2 (1.1%)	3 (1.5%)	
T1c	10 (4.1%)	14 (7.7%)	15 (7.5%)	
T2	73 (30.2%)	72 (39.8%)	84 (42.2%)	
T3	50 (20.7%)	29 (16%)	32 (16.1%)	
T4	109 (45%)	63 (34.8%)	64 (32.2%)	
Stage				0.026 ^b^
I A	5 (2.1%)	12 (6.6%)	12 (6%)	
I B	27 (11.2%)	25 (13.8%)	16 (8%)	
II A	12 (5%)	11 (6.1%)	6 (3%)	
II B	50 (20.7%)	38 (21%)	59 (29.6%)	
III	99 (40.9%)	71 (39.2%)	81 (40.7%)	
IV	49 (20.2%)	24 (13.2%)	25 (12.6%)	

^b^ Chi-square test.

**Table 4:** The variation in N lymph node involvement.

Variables	Pre-Pandemic	Pandemic	Post-Pandemic	*p*
N0	45 (33.1%)	48 (40.7%)	35 (24.8%)	0.07 ^b^
N1	52 (38.2%)	39 (33.1%)	67 (47.5%)	
N2	39 (28.7%)	31 (26.3%)	39 (27.7%)	

^b^ Chi-square test.

**Table 5:** Type of curative surgery.

Variables	Pre-Pandemic	Pandemic	Post-Pandemic	*p*
Curative				0.57 ^b^
Pancreaticoduodenectomy (Whipple procedure)	106 (84.8%)	88 (75.9%)	108 (79.4%)	
Distal pancreatectomy with splenectomy	13 (10.3%)	19 (16.1%)	16 (11.4%)	
Central pancreatectomy	2 (1.6%)	1 (0.9%)	3 (2.1%)	
Total pancreatectomy with duodenectomy	4 (3.2%)	8 (6.9%)	9 (6.4%)	
Other surgical procedures	1 (0.8%)	2 (1.7%)	4 (2.9%)	

^b^ Chi-square test.

**Table 6:** Type of palliative surgery.

Variables	Pre-Pandemic	Pandemic	Post-Pandemic	*p*
Palliative				<0.001 ^b^
Choledochoduodenostomy	74 (36.6%)	22 (15.8%)	12 (9.6%)	
Choledochojejunostomy	6 (3.0%)	8 (5.8%)	0	
Hepaticojejunostomy	20 (9.9%)	30 (21.6%)	6 (4.8%)	
Gastrojejunostomy	34 (16.8%)	22 (15.8%)	51 (40.7%)	
Other surgical procedures	68 (33.70%)	57 (41%)	56 (44.8%)	

^b^ Chi-square test.

**Table 7:** Postoperative complications.

Clavien–Dindo Classification	Pre-Pandemic	Pandemic	Post-Pandemic	*p*
Fistula				0.305 ^b^
Grade I	1 (0.9%)	0	1 (0.9%)	
Grade II	9 (8.5%)	3 (3.4%)	4 (3.7%)	
Grade IIIb	1 (0.9%)	3 (3.4%)	0	
Grade V	3 (2.8%)	2 (2.3%)	5 (4.6%)	
Hemorrhage				0.904 ^b^
Grade I	2 (1.9%)	1 (1.1%)	1 (0.9%)	
Grade II	1 (0.9%)	2 (2.3%)	1 (0.9%)	
Grade IIIa	2 (1.9%)	3 (3.4%)	5 (4.6%)	
Grade IIIb	4 (3.8%)	5 (5.7%)	4 (3.7%)	
Grade V	7 (6.6%)	4 (4.5%)	3 (2.8%)	

^b^ Chi-square test.

**Table 8:** Variations in the duration of hospital stays and surgical interventions.

Variables	Pre-Pandemic	Pandemic	Post-Pandemic	*p*
Duration of surgery (min., M ± SD)	193.36 ± 94.58	235.94 ± 103.07	239.45 ± 95.174	<0.001 ^k^
Preoperative hospitalization (days, M ± SD)	4.37 ± 3.27	3.49 ± 2.76	3.59 ± 2.41	0.006 ^a^
Postoperative hospitalization (days, M ± SD)	12.9 ± 11.7	11.3 ± 9.48	13.03 ± 8.4	0.002 ^a^
Total hospitalization (days, M ± SD)	17.27 ± 12.63	14.79 ± 9.96	16.61 ± 8.78	0.058 ^a^

M = mean; SD = standard deviation; ᵏ Kruskal–Wallis test; ^a^ ANOVA test.

## Data Availability

The datasets used and/or analyzed during the current study are available from the corresponding author upon reasonable request.
